# Costs of care during chimeric antigen receptor T-cell therapy in relapsed or refractory B-cell lymphomas

**DOI:** 10.1093/jncics/pkae059

**Published:** 2024-08-08

**Authors:** Mengyang Di, Kunal C Potnis, Jessica B Long, Iris Isufi, Francine Foss, Stuart Seropian, Cary P Gross, Scott F Huntington

**Affiliations:** Section of Hematology, Department of Internal Medicine, Yale University School of Medicine, New Haven, CT, USA; Cancer Outcomes, Public Policy and Effectiveness Research Center, Yale University, New Haven, CT, USA; Division of Hematology and Oncology, Department of Medicine, University of Washington/Fred Hutchinson Cancer Center, Seattle, WA, USA; Section of Hematology, Department of Internal Medicine, Yale University School of Medicine, New Haven, CT, USA; Cancer Outcomes, Public Policy and Effectiveness Research Center, Yale University, New Haven, CT, USA; Section of Hematology, Department of Internal Medicine, Yale University School of Medicine, New Haven, CT, USA; Section of Hematology, Department of Internal Medicine, Yale University School of Medicine, New Haven, CT, USA; Section of Hematology, Department of Internal Medicine, Yale University School of Medicine, New Haven, CT, USA; Section of Hematology, Department of Internal Medicine, Yale University School of Medicine, New Haven, CT, USA; Cancer Outcomes, Public Policy and Effectiveness Research Center, Yale University, New Haven, CT, USA; Section of Hematology, Department of Internal Medicine, Yale University School of Medicine, New Haven, CT, USA; Cancer Outcomes, Public Policy and Effectiveness Research Center, Yale University, New Haven, CT, USA

## Abstract

High upfront cost may be a barrier to adopting chimeric antigen receptor T-cell (CAR-T) therapy for relapsed or refractory B-cell lymphoma. Data on the real-world costs are limited. Using the Blue Cross Blue Shield Axis database, we evaluated 271 commercially insured patients who received CAR-T therapy for B-cell lymphoma (median age = 58 years; men = 68%; diffuse large B-cell lymphoma = 87%; inpatient CAR-T therapy = 85%). Our peri–CAR-T period of interest was from 41 days before to 154 days after CAR-T therapy index divided into seven 28-day intervals. Median total costs were $608 100 (interquartile range, IQR = $534 100-$732 800); 8.5% of patients had total costs exceeding $1 million. The median cost of CAR-T therapy products was $402 500, and the median out-of-pocket copayment was $510. Monthly costs were highest during the month of CAR-T therapy administration (median = $521 500), with median costs below $25 000 in all other 28-day intervals. Costs of CAR-T therapy use were substantial, largely driven by product acquisition. Future studies should examine the relationship between costs, access, and financial outcomes.

Chimeric antigen receptor T-cell (CAR-T) therapy can be beneficial in relapsed or refractory B-cell lymphoma (BCL), including heavily pretreated diseases, and provides the potential for durable remission (1-7). Despite encouraging efficacy, logistical challenges (8) and high upfront costs (9,10) can be barriers to adopting CAR-T therapy (11). Data on the costs and health-care resource utilization associated with CAR-T therapy in a real-world setting are limited. Further, evidence on the out-of-pocket copay expenses that patients pay is limited. To address these knowledge gaps, we performed a retrospective cohort study using a large, private insurance database.

We used the Blue Cross Blue Shield Axis database, which provides deidentified administrative claims for commercial members across the United States. We selected adult patients who had CAR-T therapy claims (Supplementary Table 1, A, available online) between January 2018 and June 2021, with continuous enrollment for a minimum of 2 calendar months before and through the calendar month of CAR-T therapy, and diffuse large B-cell lymphoma, follicular lymphoma, or mantle cell lymphoma but no acute B-cell lymphoblastic leukemia (Supplementary Table 1, B, available online). Similar to prior work (10), we required product reimbursed (or hospitalization for CAR-T therapy) cost in excess of $250 000 (an approximate amount reimbursed by the Centers for Medicare & Medicaid Services for Medicare patients) to substantiate receipt of commercial CAR-T therapy (Figure 1, A). In individuals who undergo apheresis for CAR-T therapy but never receive this commercial therapy, claims may be present for T-cell collection, but the expected high reimbursement for CAR-T products would not be present. We defined the CAR-T index as the date of CAR-T infusion or the admission date of hospitalization for CAR-T therapy if the former was not identified. The CAR-T index month (month 0) covered from 13 days before to 14 days after the CAR-T index. The peri–CAR-T period extended from 41 days before to 154 days after the CAR-T index divided into 28-day months (month ‒1 to month +5) (Figure 1, B).

**Figure 1.**
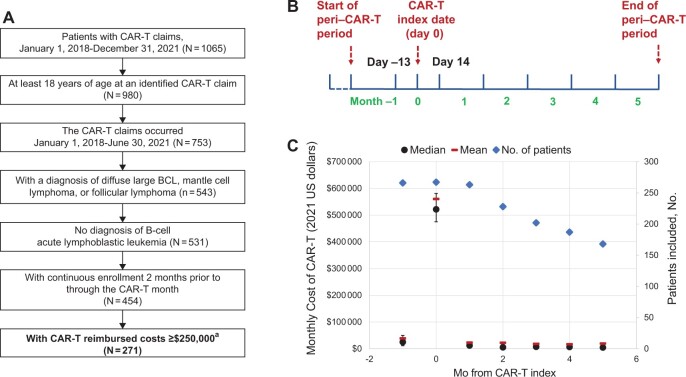
Patient selection, peri–CAR-T period, and monthly costs during the peri–CAR-T period. **A)** Flow diagram of patient selection. **B)** Illustration of the peri–CAR-T period. **C)** Average monthly costs associated with the use of CAR-T during the peri–CAR-T period. BCL = B-cell lymphoma; CAR-T = chimeric antigen receptor T-cell. ^a^Line item for CAR-T delivery or total costs of hospitalization for CAR-T therapy if the former was not available.

For health-care resource utilization, we examined hospital admission 30 days or less after the CAR-T index among patients receiving CAR-T therapy in the outpatient setting. In the overall population, we studied emergency department evaluations, hospital admissions, intensive care unit stays, physician office visits, hospice enrollment, and receipt of transplantation or systemic therapies for BCL (Supplementary Table 1, C, available online) after the CAR-T index (outpatient CAR-T therapy) or hospitalization for CAR-T therapy (inpatient delivery) and 6 months of less of the CAR-T index. We estimated length of stay, intensive care unit transfer, and death for the CAR-T hospitalization group. We studied the total cost (2021 US dollars) of care from the payer perspective based on allowed amounts for all claims during the entire peri–CAR-T period and by 28-day intervals, respectively. We estimated the costs of inpatient and outpatient care, pharmacy, and out-of-pocket copays (including deductible, copay, and coinsurance) during the same periods.

Our study cohort included 271 patients with a median age of 58 years (interquartile range, IQR = 51-62); 183 (68%) were men, 237 (87%) had diffuse large BCL, and 110 (54%) travelled 50 miles or more to receive CAR-T therapy among individuals with data on distance of travel available (n = 204). CAR-T therapy was administered in the hospital for 230 (85%) patients (Table 1). The median length of stay for individuals hospitalized for CAR-T therapy was 15 days, with 17% experiencing an intensive care unit stay and 1% dying during this admission. Post–CAR-T emergency department and hospital utilization was numerically higher in patients receiving CAR-T therapy in the outpatient setting (Table 1).

**Table 1.** Baseline characteristics, health-care resource utilization, and costs associated with administration of CAR-T therapy during the peri–CAR-T period

Baseline characteristic		Inpatient CAR-T therapy (n = 230)	Outpatient CAR-T therapy (n = 41)	Total (N = 271)
Age at CAR-T receipt, median (IQR), y		58 (51-63)	59 (51-62)	58 (51-62)
Male sex, No. (%)		152 (66)	31 (76)	183 (68)
Type of lymphoma, No. (%)				
Diffuse large B-cell lymphoma		199 (87)	38 (93)	237 (87)
Follicular lymphoma		3 (1)	1 (2)	4 (1)
Mantle-cell lymphoma		28 (12)	2 (5)	30 (11)
Travel ≥50 mi, No. (%)^a^		94 (55)	16 (50)	110 (54)
**Health-care resource utilization**		**Inpatient CAR-T therapy**	**Outpatient CAR-T therapy**	**Total**
30-d hospitalization,^b^ No. (%)		124 (54)	24 (59)	148 (55)
CAR-T hospitalization				
Intensive care unit transfer, No. (%)		47 (20)	0 (0)	47 (17)
Death, No. (%)		4 (2)	0 (0)	4 (1)
Length of stay, median (IQR), d		15 (12-20)	N/A	15 (12-20)
6-mo outcomes,^b^ No. (%)				
Emergency department visit		71 (31)	23 (56)	94 (35)
Hospitalization		106 (46)	33 (80)	139 (51)
Intensive care unit stay		47 (20)	12 (34)	59 (22)
Physician office visit		209 (91)	41 (100)	250 (92)
Hospice enrollment		21 (9)	2 (5)	23 (8)
Allogeneic stem cell transplantation		3 (1)	1 (2)	4 (1)
Autologous stem cell transplantation		2 (1)	2 (5)	4 (1)
Nonoral systemic therapy		34 (15)	7 (17)	41 (15)
Oral systemic therapy		14 (10)	2 (9)	16 (10)
**Costs, $**		**Inpatient CAR-T therapy**	**Outpatient CAR-T therapy**	**Total**
Total	No.	226	41	267
	Median (IQR)	623 300 (546 400-744 200)	584 600 (478 500-630 600)	608 100 (534 100-732 800)
	Mean (SE)	692 000 (16 600)	607 700 (34 900)	679 000 (15 100)
All hospitalizations	No.	226	33	259
	Median (IQR)	526 000 (467 100-632 900)	42 800 (17 800-122 400)	499 100 (440 100-613 600)
	Mean (SE)	570 600 (18 000)	91 100 (26 600)	509 500 (18 900)
Outpatient	No.	225	41	266
	Median (IQR)	78 000 (41 800-130 100)	502 200 (443 100-567 800)	92 100 (46 800-263 000)
	Mean (SE)	121 900 (9000)	534 300 (22 100)	185 500 (12 400)
CAR-T product^c^	No.	63	38	101
	Median (IQR)	395 300 (390 500-437 200)	407 400 (390 500-430 900)	402 500 (390 500-433 900)
	Mean (SE)	442 100 (18 700)	433 100 (20 100)	438 700 (13 900)
Hospitalization for CAR-T therapy	No.	226	0	226
	Median (IQR)	490 800 (449 000-552 700)	N/A	490 800 (449 000-552 700)
	Mean (SE)	508 500 (16 200)	N/A	508 500 (16 200)
Pharmacy^d^	No.	143	22	165
	Median (IQR)	3100 (600-17 000)	2500 (500-6800)	3000 (600-15 300)
	Mean (SE)	13 200 (2100)	7500 (2800)	12 400 (1800)
Out of pocket copay	No.	226	41	267
	Median (IQR)	540 (0-3150)	350 (0-2650)	510 (0-3150)
	Mean (SE)	1840 (160)	1660 (330)	1810 (150)

a Distance of travel was the ellipsoid distance between centroid longitude and latitude of member zip code and billing provider zip code, estimated by the “geodist” command in Stata (StataCorp, College Station, TX). Patients with unknown distance of travel were excluded from the calculation of percentage. CAR-T = Chimeric antigen receptor T cell; SE = Standard error; IQR = Interquartile range; N/A = not applicable or not available.

b Outcome events occurred within 30 days (or 6 months) after the CAR-T index for patients receiving CAR-T therapy in the outpatient setting or after hospitalization for CAR-T therapy; hospitalization of patients receiving therapy in the hospital represents readmission after their hospitalization for CAR-T therapy.

c CAR-T product cost was the allowed amount with the CAR-T outpatient claim or the inpatient claim associated with a CAR-T infusion based on Healthcare Common Procedure Coding System code.

d Not including CAR-T product costs.

The median total cost during the peri–CAR-T therapy period was $608 100 (IQR = $534 100-$732 800), with a mean (standard error, SE) of $679 000 ($15 100). Approximately 8.5% patients had a total cost exceeding $1 million. The median CAR-T product cost was $402 500 (IQR = $390 500-$433 900). The median out-of-pocket copay was $510 (IQR = $0-$3150) (Table 1). Monthly costs were highest during the CAR-T index month (median = $521 500), with the median less than $25 000 in all other 28-day months during the peri–CAR-T therapy period (Figure 1, C; Supplementary Table 2, available online). The high cost in the CAR-T index month was primarily driven by the product acquisition cost and whether other costs (eg, that associated with leukapheresis) were included but did not substantially change the total costs during the index month. The average costs were higher with inpatient delivery of CAR-T therapy, during the entire peri–CAR-T therapy period and in most 28-day months (Table 1; Supplementary Table 2, available online).

In this real-world cost analysis of CAR-T therapy for relapsed or refractory BCL—the largest, to our knowledge, we report costs and health-care resource utilization in a commercially insured population in the United States. The average costs during our 196-day peri–CAR-T therapy period were above $600 000, largely driven by product acquisition costs (median = $402 500). The average out-of-pocket copay was $510, but it did not reflect the full range of the financial burden that patients receiving CAR-T therapy face (ie, travel, caregiver, and personal expenses).

Other studies (9,10) have also focused on the real-world costs of CAR-T therapy for BCL. One study identified a population (N = 191) with heterogeneous insurance coverage (ie, 52%-73% commercial insurance) from 3 databases and reported average costs of $380 000 to $526 000 (2019 US dollars) within 3 months after CAR-T therapy (9). The difference in insurance coverage, however, made these results less comparable to the costs we report. Researchers from a pharmacy benefit management company found an average cost of $711 884 within 90 days of CAR-T therapy in a commercially insured sample with diffuse large BCL (N = 74) (10). This cost during a shorter period exceeded our estimates. Further between-study comparisons may be limited given the variations in design and lymphoma characteristics. Neither studies reported out-of-pocket copays for CAR-T use.

Our study also showed higher costs associated with inpatient CAR-T therapy compared with outpatient therapy delivery. This finding was consistent with the findings from a decision tree–based cost analysis ($454 611 inpatient vs $421 624 outpatient) (12). The savings from outpatient delivery accounted for just a small proportion of total costs, however, because of the substantial cost of CAR-T products. Thus, implementing policy changes to mitigate CAR-T product costs, such as decentralization of CAR-T manufacturing (11) or outcome-based reimbursement (13), may be needed to reduce overall CAR-T expenditures.

As the largest study on this important topic, our analysis has several strengths. We report granular data on costs of inpatient and outpatient care and pharmacy as well as monthly costs. The latter allows cross-study comparisons in similar populations, despite varied definitions or durations of the peri–CAR-T therapy period. We also estimated median out-of-pocket copays associated with CAR-T use, which had been largely unknown. In addition, our study was conducted by researchers from an academic center, which helped reduce potential bias related to conflict of interests for this type of analysis (14).

Nevertheless, our study has limitations. First, our study focused primarily on descriptive analyses given the limited sample size, which was partially the result of the relatively short interval since the first approval of CAR-T therapy. Second, CAR-T costs were estimated based on either outpatient CAR-T claims or single lines of CAR-T claims during hospitalization. Line-item claims were not available for all study participants (ie, some had total costs only for hospitalization for CAR-T therapy). Third, the lack of granular clinical and demographic data prohibit comparisons between CAR-T therapy recipients and nonrecipients as well as between patients receiving CAR-T therapy delivered in inpatient and outpatient settings. Future work should evaluate characteristics associated with nonreceipt of intended CAR-T therapy in the real-world setting. Fourth, we report out-of-pocket copays and distance of travel as potential barriers to receiving CAR-T therapy; however, we were not able to examine financial toxicities comprehensively (ie, costs of travel, lodging, and meals) (11), and the dichotomized distance may not fully reflect actual travel burdens. Furthermore, our analysis considers only out-of-pocket costs experienced by patients undergoing CAR-T therapy, with the potential for some patients facing high copays to forgo this treatment (15). Fifth, our study was based on privately insured patients; our findings on costs and out-of-pocket expenses may not be extrapolated to those relying on public insurance. Finally, switching insurance, which may not be uncommon in CAR-T administration, may partially contribute to the decreased size of our cohort during follow-up.

In our analysis examining real-world CAR-T costs, we found average total costs in excess of $600 000 for commercially insured patients with lymphoma, largely driven by substantial product acquisition costs. We found relatively low out-of-pocket expenses, but future studies should consider other patient expenses during CAR-T therapy. Policy changes will likely be needed to mitigate CAR-T product costs and improve equitable access.

## Supplementary Material

pkae059_Supplementary_Data

## Data Availability

The analyses in this study used the Blue Cross Blue Shield Axis database. Data requests may be made directly to the research department of Blue Cross Blue Shield.
