# Supplementary material for: Costs of care during chimeric antigen receptor T-cell therapy in relapsed or refractory B-cell lymphomas
Source: JNCI Cancer Spectr. 2024 Aug 8;8(4):pkae059. doi: 10.1093/jncics/pkae059 (PMC11340641; doi:10.1093/jncics/pkae059)
Supplement: pkae059_Supplementary_Data [file pkae059_supplementary_data.pdf]

Supplementary Material

Supplementary Table 1: Healthcare Common Procedure Coding System (HCPCS) codes for CAR-T claims, International Classification of Diseases (ICD) codes for lymphoma diagnosis, and HCPCS codes/generic names for lymphoma treatment

A. Codes to identify CAR-T claims		
**HCPCS**	**Description**	**Treatment Category**
0537T	Chimeric antigen receptor T-cell (CAR-T) therapy; harvesting of blood-derived T lymphocytes for development of genetically modified autologous CAR-T cells, per day	Preparation
0538T	Chimeric antigen receptor T-cell (CAR-T) therapy; preparation of blood-derived T lymphocytes for transportation (e.g., cryopreservation, storage)	Preparation
0539T	Chimeric antigen receptor T-cell (CAR-T) therapy; receipt and preparation of CAR-T cells for administration	Preparation
0540T	Chimeric antigen receptor T-cell (CAR-T) therapy; CAR-T cell administration, autologous	Infusion
Q2040	Tisagenlecleucel, up to 250 million car-positive viable t cells, including leukapheresis and dose preparation procedures, per infusion	Infusion
Q2041	Axicabtagene ciloleucel, up to 200 million autologous anti-cd19 car positive viable t cells, including leukapheresis and dose preparation procedures, per therapeutic dose	Infusion
Q2042	Tisagenlecleucel, up to 600 million car-positive viable t cells, including leukapheresis and dose preparation procedures, per therapeutic dose	Infusion
Q2053	Brexucabtagene autoleucel, up to 200 million autologous anti-cd19 car positive viable t cells, including leukapheresis and dose preparation procedures, per therapeutic dose	Infusion
Q2054	Lisocabtagene maraleucel, up to 110 million autologous anti-cd19 car-positive viable t cells, including leukapheresis and dose preparation procedures, per therapeutic dose	Infusion
C9073	Brexucabtagene autoleucel, up to 200 million autologous anti-cd19 car positive viable t cells, including leukapheresis and dose preparation procedures, per therapeutic dose	Infusion
C9076	Lisocabtagene maraleucel, up to 110 million autologous anti-cd19 car-positive viable t cells, including leukapheresis and dose preparation procedures, per therapeutic dose	Infusion
**DRG**	**Description**	
016	Autologous bone marrow transplant with CC/MCC (used for CART until 2021)	
017	Autologous bone marrow transplant without CC/MCC (used for CART until 2021)	
018	Chimeric antigen receptor (CAR) T-cell and other immunotherapies	

B. ICD codes for lymphoma diagnosis	
**Lymphoma**	**ICD codes**
Diffuse large B cell lymphoma	C83.3, C83.30-C83.39

Follicular lymphoma	C82.90-C82.99
Mantle cell lymphoma	C83.10-C83.19
B-cell acute lymphoblastic leukemia (to be excluded)	C91.0, C91.00-C91.02

C. HCPCS codes/generic names for lymphoma treatment

**IV drug**	**HCPCS**
Rituximab	J9310, J9311, J9312
Cyclophosphamide	J9070, J9080, J9090, J9091, J9092, J9093, J9094, J9095, J9096, J9097
Doxorubicin (including liposomal doxorubicin)	J9000, J9001, C9415, J9002, Q2048, Q2049, Q2050
Vincristine	J9370, J9375, J9380, J9371
Cytarabine	C9422, J9098, J9100, J9110, J9153, C9024
Bendamustine	J9033, J9034, C9423, J9036, C9042
Gemcitabine	J9201
Carboplatin	J9045
Cisplatin	J9060, J9062, C9418
Oxaliplatin	J9253
Methotrexate	J8610, J9250, J9260
Bortezomib	J9041, J9044
Etoposide	C9414, C9425, J8560, J9181, J9182
Ifosfamide	C9427, J9208
Mitoxantrone	J9293
Procarbazine	S0182
Polatuzumab vedotin	J9309
Tafasitamab	J9349
Vinorelbine	C9440, J9390
Brentuximab vedotin	J9042, C9287
Loncastuximab tesirine	C9084, J9359
Obinutuzumab	J9301, C9021
Ofatumumab	C9260, J9302
Chlorambucil	S0172
Copanlisib	C9030, J9057
**Oral drugs**	**Generic Name**
Venetoclax	Venetoclax
Ibrutinib	Ibrutinib
Acalabrutinib	Acalabrutinib
Zanubrutinib	Zanubrutinib
Lenalidomide	Lenalidomide
Tazemetastat	Tazemetastat
Umbralisib	Umbralisib
Selinexor	Selinexor

Supplementary Table 2: Monthly costs associated with the use of CAR-T during the peri-CAR-T period (2021 USD)

Month	Population	N	Cost value (2021 USD)	
			Median (IQR)	Mean (SE)
-1	Overall	266	24,800 (11,400, 49,700)	38,600 (3,100)
	Inpatient CART	226	22,900 (11,400, 51,900)	39,400 (3,500)
	Outpatient CART	40	32,300 (13,800, 43,200)	34,300 (4,400)
0	Overall	267	521,500 (475,000, 581,100)	560,900 (12,400)
	Inpatient CART	226	527,500 (484,900, 585,900)	570,700 (13,500)
	Outpatient CART	41	434,500 (407,000, 540,000)	507,000 (31,100)
1	Overall	263	12,100 (4,700, 23,500)	23,000 (2,200)
	Inpatient CART	222	12,900 (5,700, 22,900)	23,200 (2,300)
	Outpatient CART	41	8,200 (2,200, 24,900)	21,900 (5,800)
2	Overall	228	5,100 (900, 16,300)	22,100 (3,500)
	Inpatient CART	193	6,200 (1,200, 17,500)	23,800 (4,100)
	Outpatient CART	35	3,600 (400, 6,400)	12,700 (5,100)
3	Overall	202	7,100 (2,000, 18,400)	18,400 (2,100)
	Inpatient CART	168	7,000 (1,500, 19,600)	19,100 (2,300)
	Outpatient CART	34	8,600 (4,800, 13,400)	14,800 (4,200)
4	Overall	187	6,600 (1,500, 18,100)	16,800 (2,000)
	Inpatient CART	158	6,700 (1,600, 18,100)	16,800 (2,200)
	Outpatient CART	29	4,800 (1,500, 15,400)	16,800 (5,600)
5	Overall	168	4,300 (600, 17,600)	19,700 (3,800)
	Inpatient CART	142	4,400 (600, 17,500)	20,400 (4,300)
	Outpatient CART	26	1,900 (500, 17,700)	16,100 (5,400)

CART=Chimeric antigen receptor T cell therapy, USD=United States dollar, SE=Standard error, IQR=Interquartile range, NA=Not applicable
